# Detection of Genes Encoding Microbial Surface Component Recognizing Adhesive Matrix Molecules in Methicillin-Resistant *Staphylococcus aureus* Isolated from Pyoderma Patients

**DOI:** 10.3390/genes14040783

**Published:** 2023-03-24

**Authors:** Mohammed Alorabi, Uroosa Ejaz, Bahram Khan Khoso, Fakhur Uddin, Samy F. Mahmoud, Muhammad Sohail, Mona Youssef

**Affiliations:** 1 Department of Biotechnology, College of Sciences, Taif University, Taif 21944, Saudi Arabia; 2 Department of Biosciences, Faculty of Life Sciences, Shaheed Zulfikar Ali Bhutto Institute of Science and Technology, Karachi 75600, Pakistan; 3 Department of Dermatology, Jinnah Sindh Medical University, Karachi 75510, Pakistan; 4 Department of Microbiology, Basic Medical Sciences Institute (BMSI), Jinnah Postgraduate Medical Centre (JPMC), Karachi 75510, Pakistan; 5 Department of Microbiology, University of Karachi, Karachi 75270, Pakistan; 6 Department of Hepatology, Gastroenterology and Infectious Diseases, Benha Teaching Hospital, Benha 13518, Egypt

**Keywords:** bone sialoprotein-binding protein, ClfA protein, MSCRAMM, antimicrobial resistance

## Abstract

Pyoderma is a common skin infection predominantly caused by *Staphylococcus aureus.* In addition to methicillin resistance, this pathogen is resistant to many other antibiotics, which ultimately limits the available treatment options. Therefore, the present study aimed to compare the antibiotic-resistance pattern, to detect the *mecA* gene and the genes encoding microbial surface component recognizing adhesive matrix molecules (MSCRAMMs) in *S. aureus* isolates. A total of 116 strains were isolated from patients suffering with pyoderma. Disk diffusion assay was opted to perform antimicrobial susceptibility testing of the isolates. Out of the isolates tested, 23–42.2% strains appeared susceptible to benzylpenicillin, cefoxitin, ciprofloxacin and erythromycin. While linezolid was found to be the most effective anti-staphylococcal drug, followed by rifampin, chloramphenicol, clindamycin, gentamicin and ceftaroline. Out of 116 isolates, 73 (62.93%) were methicillin-resistant *S. aureus* (MRSA). Statistically significant (*p* ≤ 0.05) differences in antibiotic resistance patterns between MRSA and methicillin-susceptible *S. aureus* (MSSA) were found. A significant association of resistance to ceftaroline, rifampin, tetracycline, ciprofloxacin, clindamycin, trimethoprim–sulfamethoxazole and chloramphenicol was found in MRSA. However, no significant difference was observed between MRSA and MSSA for resistance against gentamicin, erythromycin or linezolid. All cefoxitin-resistant *S. aureus*, nonetheless, were positive for the *mecA* gene. *femA* was found in all the MRSA isolates. Among other virulence markers, *bbp* and *fnbB* were found in all the isolates, while *can* (98.3%), *clfA* and *fnbA* (99.1%) were present predominately in MRSA. Thus, this study offers an understanding of antibiotic resistance MSCRAMMs, *mecA*, and *femA* gene patterns in locally isolated strains of *S. aureus*.

## 1. Introduction

*Staphylococcus aureus* is a gram-positive bacterium that causes a wide variety of clinically significant infections. It is an important pathogen that has a major impact on human health. Infections caused by this pathogen circulated in community-acquired as well as hospital-acquired settings [1]. Although it is particularly notorious for causing skin and soft-tissue infections, it has an ability to infect nearly every organ system in the human body, often with fatal consequences [2]. Pyoderma is a cutaneous infection with primary clinical presentations of impetigo, folliculitis, furuncle, carbuncle, ecthyma, erythrasma and sycosis barbae. Indeed, pyoderma refers to any pyogenic infection of the skin caused by pus-forming pathogens; however, it is most commonly used in reference to bacterial skin infections. It is a common disorder of canine skin, which arises from impaired local defense mechanisms leading to facilitation of secondary bacterial invasion of skin [3]. Nonetheless, the invasion of the skin by the pathogen is a prerequisite to initiate pyoderma. *S. aureus* is known to produce and secrete a range of extracellular matrix proteins that enable it to interact with and colonize various host tissues, including the skin. This pathogen is well equipped with a range of extracellular matrix proteins such as the binding proteins that interact with host collagens, fibronectin and fibrinogen. Collagen is a major component of connective tissues, and *S. aureus* can bind with it to penetrate and colonize deeper tissues. Fibronectin, a protein found in the extracellular matrix of many tissues, acts as a binding site for *S. aureus* and facilitates adherence and colonization of the pathogen to various host tissues. While activation of fibrinogen after having an interaction with the surface protein of *S. aureus* initiates blood-clotting pathways, *S. aureus* can evade the immune system by using these clots and can establish infections. Overall, the ability of *S. aureus* to produce and utilize extracellular matrix proteins is an important factor in its pathogenesis and ability to cause infections [4,5]. Previously, microbial surface components recognizing adhesive matrix molecules (MSCRAMMs) have been described for their capacity to cling to the extracellular matrix of the host. The proteins harbor a common and typical motif, LPXTG, that allows MSCRAMMs to remain attached to the peptidoglycan of the cell wall [6]. There can be 20 different potential MSCRAMMs that are covalently anchored to peptidoglycan [6]. These MSCRAMMs can be classified into different subfamilies based on their structural features and ligand specificity. The ability of *S. aureus* to express multiple MSCRAMMs is thought to be an important factor in its virulence and ability to cause infections. The diverse array of MSCRAMMS enable the organism to interact with host tissues in a variety of ways that ultimately assist the pathogen in evading the immune cells [5]. Since all the MSCRAMMs with a common structural organization are responsible for the binding to host-specific ligands, therefore, detection of genes encoding these molecules can assist in establishing the virulent nature of *S. aureus.* Hence, these representative genes provide insights into understanding evolutionary changes within the bacterium for population studies instead of analyzing its whole genome. The strains of *S. aureus* lacking these genes are found to be less virulent [7]. Among the 20 MSCRAMMs, fibronectin-binding protein A and B (FnBPA and FnBPB) are more profound and have been routinely detected in *S. aureus*. In fact, both of these proteins have been reported in most the *S. aureus* strains [4]. Furthermore, the clumping factors A and B (ClfA and ClfB) also bind to the fibrinogen (Fg) region and assist the pathogen in invasion [6]. Particularly, clumping factor A (ClfA) is the main fibrinogen-binding protein in *S. aureus* that is responsible for clumping the pathogen with plasma [8]. Together, these proteins promote adhesion and assist *S. aureus* in thriving in the bloodstream; hence, the pathogen can cause abscesses in skin and in internal organs [9].

In addition to expressing a variety of virulence factors, *S. aureus* also harbors many genetic determinants encoding resistance to various classes of antibiotics, including β–lactams, macrolides, aminoglycosides and fluoroquinolones. Methicillin-resistant *S. aureus* (MRSA) strains are well-recognized antibiotic-resistant bacteria posing threats to the public healthcare system as well as communities and have become a big challenge for clinicians [1]. Community-associated MRSA infections (CA-MRSA) usually affect healthy individuals with no underlying health problems, while healthcare-associated MRSA (HA-MRSA) infections occur in patients who are hospitalized or receive medical care in healthcare facilities. MRSA strains have also been emerged as pandemic clones with high virulence and are more resistant to antimicrobials [2]. MRSA strains have been reported to carry the *mecA* gene that encodes a penicillin-binding protein (PBP2a) and confers resistance to many β–lactam antibiotics, except for ceftaroline and ceftobiprole [8,9]. The staphylococcal cassette chromosome mec and *SCCmec* consist of three structural elements, including *ccr* complex, *mec* complex, and joining regions. The complex also encompasses the regulatory genes and associated insertion sequences [9,10].

Strains causing CA-MRSA are strappingly associated with skin and soft tissue infections in healthy and younger populations [11,12]. Such community-acquired pathogens have long been considered susceptible to ciprofloxacin, levofloxacin, clindamycin, chloramphenicol and to most frequently used traditional antibiotics. CA-MRSA strains have been found to harbor *SCCmec* types IV and V [13]. Nonetheless, the PBP2a production alone cannot explain the overall diversity of resistance among isolates. Some additional factors are also associated with the expression of methicillin resistance. Among those additional factors, the essential methicillin (*fem*) gene is a complex, *femABX*, where *femAB* encodes proteins containing the pentaglycine interpeptide bridge. *femA* was first described in this family and was linked to the increased resistance in MRSA. *femA* encodes a precursor protein that is essential for the synthesis of cell–wall peptidoglycan. It is a transmembrane protein that is involved in the transfer of a peptide chain to lipid II, which is a key intermediate in peptidoglycan biosynthesis. Previously, *femA* has been used as a marker for the identification of *S. aureus* as it is highly conserved among *S. aureus* strains and absent in most other bacterial species [14].

Although HA-MRSA and CA-MRSA strains may have different antimicrobial susceptibility patterns [15], both are now considered significant hazards to human health in the context of infectious diseases [16]. Understanding their respective distribution in the population is crucial for their treatment and management. Indeed, there is a lack of understanding of the prevalence of MRSA infections and common local MRSA variants. Therefore, this study was planned to investigate the patterns of resistance and virulence genes in clinical isolates of MRSA from pyoderma patients.

## 2. Materials and Methods

### 2.1. Isolation of MRSA Strains

This cross-sectional study was conducted during the period of July 2022 to December 2022 during which 116 *S. aureus* strains were isolated from the pus specimens of pyoderma suspected cases. The pus swabs were collected from the affected areas of clinically diagnosed cases of pyoderma by the dermatologist. The patients having a history of recent surgery, hospitalization, postoperative wound, burn infections and non-purulent skin infections were excluded from the sampling. The samples were collected from the outpatient department (OPD) of Dermatology, Jinnah Post Graduate Medical Centre, transported to the laboratory and inoculated on Blood Agar and MacConkey agar plates (Oxoid, Basingstoke, UK). *S. aureus* isolates were identified by the routine cultural characteristics and biochemical tests, including mannitol fermentation, coagulase, catalase and novobiocin susceptibility. The provisionally diagnosed *S. aureus* isolates were further confirmed by the Microbact™ Staph 12S system (Remel, Fremont, CA, USA) in accordance with the manufacturer’s instructions.

### 2.2. Antibiotic Susceptibility Testing

Antibiotic susceptibility tests (AST) were conducted as per guidelines and breakpoints established by the Clinical and Laboratory Standards Institute (CLSI), 2022. Twelve anti-Staphylococcal antibiotics discs (Oxid, Basingstoke, UK) including, penicillin, cefoxitin, ceftaroline, clindamycin, chloramphenicol, trimethoprim/sulfamethoxazole, erythromycin, tetracycline, gentamicin, ciprofloxacin, rifampin and linezolid were used. The MRSA isolates were phenotypically detected using the cefoxitin disc (30 µg; Oxoid, Basingstoke, UK) in accordance with the CLSI guidelines. The zone of inhibition around the cefoxitin disc was measured and interpreted using the CLSI breakpoints, i.e., susceptible ≥ 22 mm and resistant ≤ 21 mm. *S. aureus* (ATCC 25923) and MRSA (ATCC 33592) strains were used as the control strains for AST and MRSA detection, respectively.

### 2.3. Amplification of MSCRAMMs

Amplification of virulence markers of all phenotypically isolated MRSA strains was performed using PCR. An isolated colony was transferred to the phosphate buffer saline (300 µL) and heated at 95 °C for 25 min, after which the tube was cooled on ice for 5 min and then placed in a freezer for 5 min. The cell suspension was then centrifuged at 12,000 rpm for 20 min. Supernatant was carefully collected and was used as an extracted DNA sample. Primer sequences and PCR conditions are mentioned in Table S1 and Table S2, respectively. The *femA* and *mecA* genes and MSCRAMMs markers including *bbp*, *eno*, *fnbA*, *fnbB*, *fib*, *clfA*, and *clfB* were detected by visualizing the PCR products on agarose gel.

### 2.4. Statistical Analysis

The data was initially entered in the excel file and then imported to the Statistical Package for Social Science (SPSS) version 22. The qualitative variables were presented in simple frequencies. The significance and association of different variables were evaluated using Chi-square at 95% confidence limit, considering *p* ≤ 0.05 as significant.

## 3. Results and Discussion

MRSA poses a challenge for treatment options; therefore, tracking the prevalence and dissemination of such isolates is an important step in adapting infection-control measures [17]. Many reports on antibiotic resistance in MRSA have been published. However, the genotypic characterization of virulence markers in MRSA isolated from pyoderma has not been frequently reported, particularly from developing countries. Therefore, the present work is one of the few sets of data that especially emphasizes the prevalence of MRSA in skin infections with virulent factors such as MSCRAMMs genes.

**Figure 1:**
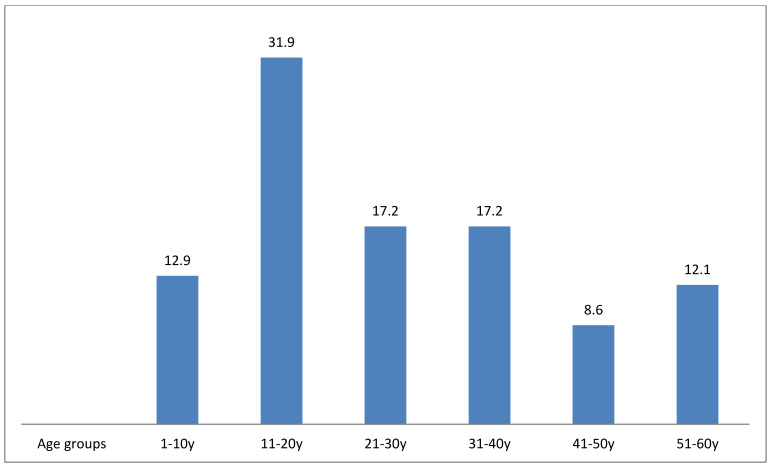
Distribution of study subjects according to the age group.

In this study, the participants were categorized into six age groups. Results showed that pyoderma cases were more frequent in patients of ages 11–20 years (Figure 1). This finding is in line with the results reported by Swathi et al. [18]. The observation is attributed to the outdoor and reckless activities of youngsters with poor hygiene leading to a higher risk of trauma [19]. This observation was further affirmed by the fact that a majority of the patients were male (81; 69.8%), as in this society, young girls rarely have extensive outdoor activities. An earlier report also corroborated the male preponderance for pyoderma cases [18]. Contrarily, a study from India reported the female preponderance [20] that can be understood by considering the different societal norms in the two countries. In this study, most patients (100/116; 86.20%) had localized infections in just a part of the body. Primary pyoderma was more frequent (89; 76.72%) than secondary pyoderma (27; 23.28%). The higher prevalence of primary pyoderma was also reported in a previous report, which strengthens the findings of this study [20]. The detailed history of patients revealed that the majority of patients (97; 83.62%) never visited the clinical laboratories for the pus culture and sensitivity. Patients’ previous drug histories showed that many patients were taking amoxicillin–clavulanic and trimethoprim–sulfamethoxazole (23.3%), followed by (16.4%) trimethoprim–sulfamethoxazole and cefaclor (Table 1).

In the present study, *S. aureus* isolates exhibited decreased (23.3–53.4%) susceptibility to penicillin, cefoxitin, erythromycin, ciprofloxacin, tetracycline and trimethoprim–sulfamethoxazole (Table 2). Nonetheless, all the isolates appeared susceptible to linezolid, which was in line with the findings of earlier studies [19,20]. Linezolid-resistant *Staphylococcus* isolates have not been frequently reported. An earlier study from Iran reported 4.1% of linezolid-resistant *S. aureus* from the hospitalized patients [21]. The high cost of antibiotics such as linezolid limits their use, particularly, for outdoor patients and hence the resistance to such drugs in community-acquired pathogens remains at a lower level. Moreover, the possibility of the spread of resistant clones in hospital settings cannot be ignored.

Ceftaroline appeared effective against most of the isolates (81%), including MRSA. Indeed, this fifth-generation cephalosporin has been approved by the FDA as an anti-MRSA drug for the skin- and soft tissue-complicated infections and community-acquired pneumonia. Its effectiveness lies in its higher affinity for PBP2a than the other traditional β–lactam antibiotics [17]. In contrast to our findings, a former study from Korea reported a higher resistance (44%) among the MRSA isolates to ceftaroline. This variation may be due to the presence of MRSA ST5, which carried a substitution of N146K in nPBD and two substitutions of L357I and I563T in PBD, which were identified in South Korea as a local ceftaroline-resistant clone [22]. Nonetheless, more studies are required to elucidate the clonal complex typing and their clone epidemiology.

**Figure 2:**
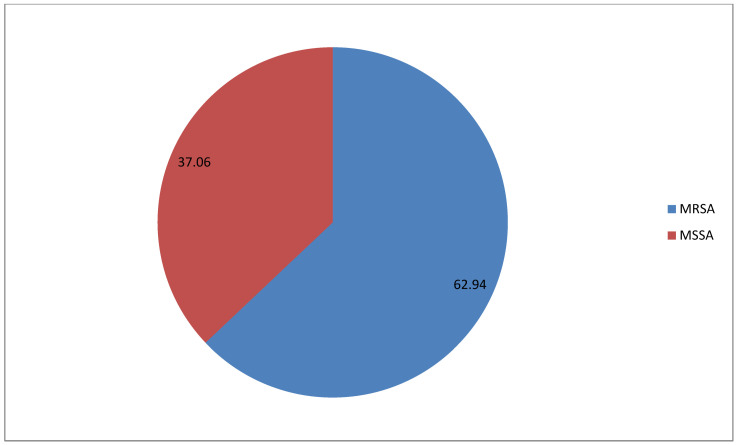
Prevalence of MRSA and MSSA in pyoderma cases.

The prevalence of MRSA (62.93%) as determined in the present study (Figure 2) differed from the reports regarding other geographic regions, including North America (>98.4%), Latin America (83.3%) and the Asia/South Pacific countries (78.8–83%) [17]. Indeed, different clones (ST239, ST72/IV or ST5/II strains) circulate in different geographic regions [17]. In addition to almost all β–lactams, MRSA strains frequently exhibit resistance to antimicrobial agents from other classes, including erythromycin, azithromycin, lincosamides, gentamicin, amikacin, glycopeptides, oxazolidinones and quinolones [16]. In the present study, resistance to non-β–lactam antibiotics, including gentamicin and erythromycin, was slightly higher in MRSA than in MSSA isolates. However, this finding was found to be statistically insignificant with *p* values higher than 0.05. Moreover, there was a significant difference in the susceptibility pattern of rifampin, tetracycline, ciprofloxacin, clindamycin and chloramphenicol in MRSA and MSSA (Table 3). Nevertheless, Qodrati et al. [23] reported similar findings except the pattern for erythromycin susceptibility that may be linked with the collection of MRSA isolates from the hospitalized patients. Nonetheless, the effectiveness of these antibiotics along with linezolid that exhibited good inhibitory activity is also subject to its cost, which can be a determining factor in many developing countries [20,22]. The availability of antibiotics as over-the-counter drugs hampers resistance in low-income regions, particularly in community-acquired infections; nevertheless, the high cost of antibiotics limits its use and, hence, a lower resistance is observed for such drugs.

**Figure 3:**
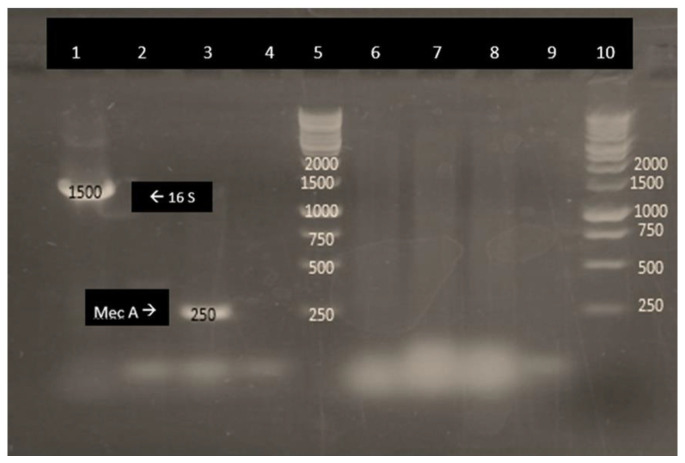
PCR product of the *mecA* gene. Lane 1 shows 16 S rDNA PCR product. Lane 2 presents *mecA* gene amplification from MRSA isolates while 3, 4, 6, 7, 8, 9 present results from Methicillin sensitive strains. Lanes 5 and 10 have ladder of 1 kb (Promega, Madison, WI, USA).

In the present study, the phenotypic data showed that 73 isolates (62.93%) were MRSA (Table 4). The finding was also affirmed by the amplification of the *mecA* gene (Figure 3). The prevalence of MRSA reported here is higher than that reported earlier (54.1%) from different cities of Pakistan [24]. The MRSA nature of the isolates was confirmed by detecting the resistance against cefoxitin and through PCR by amplifying the *mecA* gene. The method based on the resistance against cefoxitin was found to exhibit good sensitivity and specificity in comparison to the PCR. All cefoxitin-resistant *S. aureus*, nonetheless, were positive for the *mecA* gene. Indeed, finding the *mecA* gene is the major evidence for the detection of MRSA isolates, which has been endorsed by many workers [24,25]. MRSA strains that are community-associated have become a significant source of infection in people who have never visited the healthcare system. Our findings suggested the higher burden (62.92%) of the MRSA in contrast to the study conducted by Ahmed and Asrar [26] where a much lower prevalence was observed (27.8%) while studying community-acquired infections. Since the report [27] appeared eight years ago, the emergence of antibiotic resistance is an ever-increasing phenomenon, the higher prevalence in this study can be associated with the evolution of more resistant isolates and the spread of a specific antibiotic-resistance clone or several clones. With the higher population rate of the country and dwindling healthcare facilities, more people access antibiotics through over-the-counter sales and, hence, resistance is increased exponentially. Age, gender, socioeconomic level, family size, and comorbid conditions such as diabetes may also have acted as confounding factors. The recent COVID-19 epidemic caused indiscriminate use of antibiotics to prevent otherwise self-managed respiratory problems and has augmented the severity of the situation. Thus, a systemic study involving large data sets is required to understand the main reasons. This increase in prevalence indicates an urgency to identify the control measures for the spread and upsurge of CA-MRSA. Furthermore, various studies have summarized irrational antibiotic use, overcrowded living places, high nasal colonization and a higher incidence of diabetes as determining factors of increasing antibiotic resistance in community-acquired pathogens [26].

The level of methicillin resistance is mainly influenced by the Fem (factor essential for methicillin resistance) proteins encoded by *femA* and *femB* [28]. Both genes are essential for the formation of pentaglycine, which is required for the formation of peptidoglycan. The absence of these genes results in greater susceptibility to β–lactam antibiotics [28]. In this study, all the MSSA and MRSA isolates (116) were found positive for the *femA* gene. Meng et al. [10] have also reported the ubiquitous presence of the *femA* gene in *S. aureus* isolates regardless of their resistance to methicillin. *femA* has been described as a regulatory marker for methicillin resistance [23,28]. *femA* cannot confer resistance to the bacteria if other markers are absent. The role of other factors contributing resistance in MRSA has been discussed by Zuniga et al. [28].

The frequencies of individual MSCRAMM genes among isolates are presented in Table 5. Indeed, *S. aureus* can express a wide variety of MSCRAMMs genes, although some of those genes are evaluated in this study based on their main role in pathogenicity [6]. The frequencies of individual MSCRAMM genes among *S. aureus* isolates can vary depending on geographical location, the population being studied, and the type of infection being analyzed. The prevalence of *clfA*, *fnbA*, *eno*, *fib*, and *cna* genes in all isolates were 115 (99.1%), 115 (99.1%), 86 (74.15%), 85 (73.3%) and 114 (98.3%), respectively. All the MRSA isolates were positive for *bbp* and *fnbB* genes. In addition, the MRSA strains that carried four MSCRAMMs determinants, including *fib*, *cna*, *eno* and *fnbB*, should be carefully considered since all these strains were also MDR. Results showed that a majority of the MRSA isolates harbored genes encoding binding factors. The high prevalence of genes encoding adhesive protein indicates the persistence of these strains after colonization and subsequent infection. The *fnbB* gene encoding fibronectin-binding protein B was detected in all the isolates. In a previous study, all those MRSA strains that carried the *fnbB* gene were considered as the most virulent strains with multiple resistance phenotypes [1]. In general, regardless of the source, we and others have discovered that MSCRAMM genes are shared by *S. aureus* isolates from many genetic origins [6]. However, further studies are needed to give precise information about the virulence factors of these strains.

There are certain limitations associated with this work. First, it is a single-center study, yet the sampling site, Jinnah Postgraduate Medical Center, is a tertiary care hospital and most of the cases are referred from different parts of the country. The samples were collected from the outdoor patients of pyoderma; therefore, a comparison cannot be drawn between community-acquired and hospital-acquired isolates. Moreover, the study did not investigate the ability of the isolates to exhibit phenotypic characteristics of biofilm formation and host-binding capabilities. Hence, the study involving larger data sets from multiple centers investigating the MSCRAMMs determinants in community-acquired and hospital-acquired isolates of MRSA and MSSA can affirm the hypothesis regarding evolution of the resistant clones.

## 4. Conclusions

The frequency of methicillin-resistant *S. aureus* (MRSA) in the community cases of pyoderma were comparatively higher than the previous studies. Furthermore, MRSA isolates were frequently resistant to most of the non-β–lactam antimicrobial agents, including, tetracycline, erythromycin, gentamicin, trimethoprim–sulfamethoxazole and ciprofloxacin. The linezolid and rifampin were found to be the most effective drugs against all isolates of *S. aureus*. *S. aureus* isolates were found to invariably harbor the *femA* marker, while *femB* was found only in MRSA. Among tested microbial surface component-recognizing adhesive matrix molecules in MRSA, most of the genes were detected in these isolates. The study witnessed a surge in MRSA prevalence from pyoderma patients; hence, a judicious strategy needs to be formulated to curb this ever-increasing resistance. Therefore, it is advisable for the dermatologist to confirm the MRSA nature of the etiological agent and its susceptibility prior to the initiation of antibiotic treatment.

## Figures and Tables

**Table 1:** Treatment history of patients included in this study at the time of collection of specimens (n = 116).

Antimicrobial Agent	Frequency	Percent
AMC	11	9.5
AMC, SXT + Antifungal	1	0.9
AMC + FD	1	0.9
AMC + SXT	27	23.3
AMP, SXT	2	1.7
Antifungal	3	2.6
CD	4	3.4
CF	11	9.5
CF + VA	2	1.7
CF + SXT	19	16.4
CRO/SXT	5	4.3
LNZ	1	0.9
No	16	13.8
Steroids	2	1.7
SXT	9	7.8
VA, CF	1	0.9
Total	116	100.0

AMC: amoxicillin–clavulanic acid, AMP: ampicillin, CD: clindamycin, CF: Cefaclor, CRO: ceftriaxone, LNZ: linezolid, SXT: trimethoprim–sulfamethoxazole, VA: Vancomycin.

**Table 2:** Susceptibility pattern of *S. aureus* (n = 116).

Antibiotics	Number of Sensitive Strains	Percent
Penicillin	27	23.3
Cefoxitin	43	37.06
Ceftaroline	94	81.0
Gentamicin	96	82.8
Erythromycin	49	42.2
Tetracycline	53	45.7
Ciprofloxacin	44	37.9
Clindamycin	103	88.8
Trimethoprim–sulfamethoxazole	62	53.4
Chloramphenicol	103	88.8
Linezolid	116	100.0
Rifampin	108	93.1

**Table 3:** Comparative analysis of non-β–lactam antibiotics susceptibility patterns of MSSA with MRSA isolates.

Antibiotic	Susceptibility	MSSA (43)	MRSA (73)	*p*-Value
Ceftaroline	R	3 (6.97)	19 (26.03)	0.01
	S	40 (93.03)	54 (73.97)	
Gentamicin	R	5 (11.62)	15 (20.55)	0.2
	S	38 (88.38)	58 (79.45)	
Erythromycin	R	21 (48.84)	46 (63.01)	0.1
	S	22 (51.16)	27 (36.97)	
Linezolid *	R	-	-	-
	S	43 (100)	73 (100)	
Rifampin	R	-	8 (10.96)	0.02
	S	43 (100)	65 (89.04)	
Tetracycline	R	18 (41.86)	45 (61.64)	0.03
	S	25 (58.14)	28 (38.36)	
Ciprofloxacin	R	14 (32.56)	58 (79.45)	0.00
	S	29 (67.44)	15 (20.55)	
Clindamycin	R	-	13 (17.81)	0.00
	S	43 (100)	60 (82.19)	
Trimethoprim sulfamethoxazole	R	9 (20.93)	45 (61.64)	0.00
	S	34 (79.07)	28 (38.36)	
Chloramphenicol	R	-	13 (17.81)	0.00
	S	43 (100)	60 (82.19)	

The results are significant at *p* < 0.05: * No statistics are computed because linezolid is constant.

**Table 4:** Prevalence of *mecA* gene in *S. aureus* isolates.

Prevalence	Frequency	Percent	Valid Percent
Negative	43	37.07	37.07
Positive	73	62.93	62.93
Total	116	100.0	100.0

**Table 5:** MSCRAMMs positivity rate in *S. aureus* isolates.

MSCRAMMs	Number	Percent
*clfA*	115	99.1
*bbp*	116	100.0
*fnbA*	115	99.1
*fnbB*	116	100.0
*eno*	86	74.1
*fib*	85	73.3
*cna*	114	98.3

## Data Availability

The data associated with this manuscript can be obtained from the corresponding author upon a reasonable request.

## Supplementary Materials

The following supporting information can be downloaded at: https://www.mdpi.com/article/10.3390/genes14040783/s1, Table S1: Detail of Primers; Table S2: Conditions for amplification of genes.
