# Supplementary material for: Detection of Genes Encoding Microbial Surface Component Recognizing Adhesive Matrix Molecules in Methicillin-Resistant Staphylococcus aureus Isolated from Pyoderma Patients
Source: Genes (Basel). 2023 Mar 24;14(4):783. doi: 10.3390/genes14040783 (PMC10137381; doi:10.3390/genes14040783)
Supplement: Supplementary file 1 [file genes-14-00783-s001.zip › genes-2276663-supplementary.pdf]

Supplementary File

Table S1 Detail of Primers.

**S. No**	**Name of primer**	**Primer direction**	**Sequence**	**Size of product (bp)**
1.	*mec A*	Forward	5’TGCTATCCACCCTCAAACAGG3’	286
		Reverse	5’AACGTTGTAACCACCCCAAGA3’	
2.	*fem A*	Forward	5’TGCTATCCACCCTCAAACAGG3’	450
		Reverse	5’AACGTTGTAACCACCCCAAGA3’	
3.	*bbp*	Forward	5'AACTACATCTAGTACTCAACAACAG3'	575
		Reverse	5'ATGTGCTTGAATAACACCATCATCT3'	
4.	*cna*	Forward	5'GTCAAGCAGTTATTAACACCAGAC3'	423
		Reverse	5'AATCAGTAATTGCACTTTGTCCACTG3'	
5.	*eno*	Forward	5'ACGTGCAGCAGCTGACT3'	302
		Reverse	5'CAACAGCATYCTTCAGTACCTTC3'	
6.	*fnbA*	Forward	5'GTGAAGTTTTAGAAGGTGGAAAGATTAG3'	643
		Reverse	5'GCTCTTGTAAGACCATTTTTCTTCAC3'	
7.	*fnbB*	Forward	5'GTAACAGCTAATGGTCGAATTGATACT3'	524
		Reverse	5'CAAGTTCGATAGGAGTACTATGTTC3'	
8.	*fib*	Forward	5'CTACAACTACAATTGCCGTCAACAG3'	404
		Reverse	5'GCTCTTGTAAGACCATTTTCTTCAC3'	
9.	*clfA*	Forward	5'ATTGGCGTGGCTTCAGTGCT3'	292
		Reverse	5'CGTTTCTTCCGTAGTTGCATTTG3'	

Table S2. Conditions for amplification of genes.

Gene	PCR conditions						
	**Hot start**	**Denaturation**	**Annealing**	**Elongation**	**Extension**	**Storage**	**No. of cycles**
mecA	97°C for 6 min	92°C for 30 s	55°C for 30 s	72°C for 45 s	72°C for 10 min	4°C	30
femA	94°C for 5 min	94°C for 30 s	55°C for 40 s	72°C for 50 s	72°C for 10 min	4°C	40
MSCRAMMs genes	94 °C for 5 min	94°C for 1 min	55°C for 1 min	72°C for 1 min	72°C for 10 min	4°C	25
